# Spatiotemporal Distribution of Hand, Foot, and Mouth Disease and the Influence of Air Pollutants and Socioeconomic Factors on Incidence in Fujian, China

**DOI:** 10.3390/tropicalmed10070188

**Published:** 2025-07-03

**Authors:** Meirong Zhan, Shaojian Cai, Zhonghang Xie, Senshuang Zheng, Zhengqiang Huang, Jianming Ou, Shenggen Wu

**Affiliations:** Fujian Provincial Center for Disease Control and Prevention, Fuzhou 350001, China; zmr@fjcdc.com.cn (M.Z.); csj@fjcdc.com.cn (S.C.); xiaokang@fjcdc.com.cn (Z.X.); szheng@fjcdc.com.cn (S.Z.); hzq@fjcdc.com.cn (Z.H.); ojm@fjcdc.com.cn (J.O.)

**Keywords:** hand, foot, and mouth disease (HFMD), spatiotemporal characteristics, air pollutants, socioeconomic factors, influence

## Abstract

Background: Hand, foot, and mouth disease (HFMD) typically exhibits spatiotemporal clustering. This study aimed to analyze the spatiotemporal heterogeneity of HFMD in Fujian Province, China, and to identify the associations of air pollutants and socioeconomic factors with the incidence. Methods: Daily reported HFMD case data, daily air pollutant data, and socioeconomic data in Fujian Province from 2014 to 2023 were collected for analysis. A descriptive analysis was used to describe the epidemiological trends of HFMD. Spatial autocorrelation analysis was applied to explore the spatiotemporal clustering characteristics. The associations between risk factors and HFMD incidence were evaluated using the generalized additive model (GAM). Results: HFMD incidence in Fujian has decreased since 2019, and the peak in each year occurred between May and June. Distinct high–high and low–low clustering areas were identified. The cumulative exposure–response curves for SO_2_, NO_2_, and CO showed a monotonically increasing trend, with relative risks (RRs) < 1 at concentrations lower than the median levels (SO_2_ ≈ 4 μg/m^3^, NO_2_ ≈ 16 μg/m^3^, CO ≈ 1 mg/m^3^). In contrast, the curves for O_3_ and PM_2.5_ showed a decreasing trend, with RR < 1 at concentrations above the median levels (O_3_ ≈ 55 μg/m^3^, PM_2.5_ ≈ 20 μg/m^3^). Among socioeconomic factors, only the proportion of the population under 15 years old was found to be associated with HFMD incidence. Conclusions: HFMD incidence in Fujian exhibited distinct spatiotemporal clustering. The incidence was associated with the concentrations of air pollutants. Targeted interventions should be implemented in high-risk areas to mitigate HFMD transmission, with particular attention given to the environmental and demographic factors.

## 1. Introduction

Hand, foot, and mouth disease (HFMD) is a widespread viral illness caused by enteroviruses, primarily Coxsackievirus A16 (CV-A16) and Enterovirus A71 (EV-A71) [1]. The primary modes of transmission involve direct contact, respiratory droplets, and fecal–oral transmission. HFMD predominantly affects children under 5 years old, with typical symptoms including rashes, blisters, and ulcers on the hands, feet, and oral mucosa. A small proportion of cases may develop severe complications, such as myocarditis, pulmonary edema, aseptic meningitis, and encephalitis, which can be life-threatening [2,3]. In recent years, HFMD outbreaks have frequently occurred in the Asia–Pacific region [4,5,6], posing a threat to the health of children and adolescents and contributing to a considerable disease burden [3,7]. Since 2010, HFMD has consistently been the most common reported notifiable infectious disease in China [8]. To date, there is no specific antiviral treatment for HFMD, and it remains a public health concern.

With the rapid industrialization and urbanization in China over the past few decades, air pollution has increased substantially. In 2016, the average concentrations of PM_2.5_ (47 μg/m^3^) and PM_10_ (82 μg/m^3^) across 338 cities in China significantly exceeded the limits recommended by the World Health Organization (WHO) air quality guidelines. Air pollutants constitute a complex and heterogeneous mixture of particulate matter (PM) and gaseous pollutants, including carbon monoxide (CO), nitrogen oxides (NOx), ozone (O_3_), and sulfur oxides (SOx). According to the WHO, nearly 99% of the global population was exposed to air pollution levels above WHO safety limits in 2022 [9]. The Global Burden of Disease (GBD) 2021 showed PM pollution as the leading environmental risk factor for disease burden worldwide, accounting for 80% (95% UI: 67–94) of disability-adjusted life years (DALYs) [10]. The State of Global Air 2024 (SoGA) report revealed that air pollution was the second leading risk factor for mortality in 2021, contributing to an estimated 8.1 million deaths globally. More than 700,000 deaths among children under five years old were related to air pollution [11].

Given that HFMD primarily affects children under five, this trend has prompted research on the association between air pollution and HFMD. Peng et al. [12] found that both extremely high and extremely low concentrations of PM_10_, O_3_, SO_2_, and CO increased HFMD risk. Zhong et al. [13] demonstrated that improving air quality, particularly by reducing PM_2.5_ and PM_10_ levels, mitigated the risk of HFMD outbreaks. Yu et al. [14] reported that low O_3_ concentration was linked to elevated HFMD incidence, and low PM_2.5_ and high O_3_ appeared to have protective effects. Huang et al. [15,16] found no significant association between PM_10_ and overall HFMD incidence, but their subsequent study in 2019 identified PM_10_ as a risk factor for HFMD incidence, specifically among girls. These inconsistent findings may be attributed to spatiotemporal variations, autocorrelation in disease incidence, and the exclusion of potential confounders. In addition to environmental factors, socioeconomic factors also play a critical role in HFMD transmission. Urbanization rate, gross domestic product (GDP) per capita, population density, birth rate, and hospital bed availability have all been shown to influence HFMD incidence [17,18,19]. However, the results of different studies vary. Jiang et al. [18] found that regions with a high urbanization rate present a higher overall risk of HFMD, while population density and the number of hospital beds transitioned from being risk factors to protective factors once exceeding certain thresholds. In contrast, the studies by Li et al. [20] and Gou et al. [21] showed a unidirectional positive association between HFMD incidence and both population density and per capita GDP. Therefore, the impact of social factors on HFMD remains inconclusive and may also vary across regions [22].

Understanding the environmental and socioeconomic factors associated with HFMD incidence is essential for developing targeted prevention and control measures to mitigate potential outbreaks. Fujian Province was selected as the study area because Fujian is one of the hotspots for HFMD incidence in China, with consistently high cases reported in national surveillance [20,23]. Additionally, the province faces relatively severe air pollution issues, and its complex topography may result in distinct spatiotemporal epidemiological patterns of the disease. This study focused on the epidemiological characteristics of HFMD in Fujian Province, aiming to explore the spatiotemporal distribution patterns from both temporal and spatial perspectives and to identify the effects of environmental and socioeconomic determinants on HFMD risk. The findings might provide evidence-based insights to support public health decision-making in the prevention and control of HFMD.

## 2. Materials and Methods

### 2.1. Study Area

**Figure 1:**
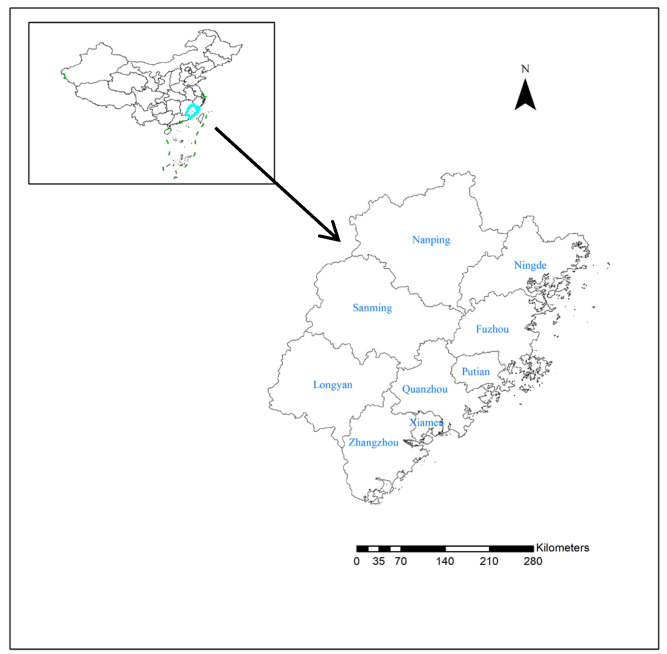
Study area.

Fujian Province is located along the southeastern coast of China, spanning 23°31′–28°18′ N latitude and 115°50′–120°43′ E longitude. Fujian administers 9 prefecture-level cities and the Pingtan Comprehensive Experimental Zone. It covers a land area of 124,000 km^2^ and a maritime area of 136,000 km^2^, with a population of approximately 41.83 million. Fujian’s terrain is predominantly composed of mountains and hills, with two major mountain ranges in the western and central areas forming the topographical backbone of the province. Numerous branches extend outward in all directions, creating a crisscrossing pattern of peaks and ridges. Hills are widely distributed along the outer mountain areas and coastal zones. Fujian has a typical subtropical climate, characterized by mild winters, relatively moderate summers, and abundant rainfall, which are conducive to the transmission of HFMD virus (Figure 1).

### 2.2. Data Collection

Daily reported HFMD case data from 1 January 2014 to 31 December 2023 were obtained from the Surveillance Reporting Management System of the China Disease Prevention and Control Information System. Corresponding daily air pollution data, including PM_2.5_, PM_10_, O_3_, SO_2_, CO, and NO_2_, were retrieved from the Resource and Environmental Science Data Platform. Annual socioeconomic data, including urbanization rate (%), GDP per capita, the number of hospital beds per 100,000 population, population density, and the proportion of population aged < 15 years, were collected from Municipal Statistical Yearbooks and Statistical Bulletins of National Economic and Social Development. Air pollutant monitoring stations in Fujian Province have been achieving full provincial coverage since 2014, and relatively complete pollutant monitoring data can be obtained. Therefore, the period from 2014 to 2023 was selected as the study period. There were no essential changes in the surveillance or diagnostic criteria during this time.

### 2.3. Statistical Analysis

The monthly number of reported cases from 2014 to 2023 was aggregated to illustrate the temporal trend of HFMD incidence in Fujian Province, and annual data were used to present the pattern of pathogen variation.

#### 2.3.1. Spatial Autocorrelation Analysis

Global and local spatial autocorrelation analyses of HFMD incidence in Fujian Province from 2014 to 2023 were conducted at the county level by year. The global Moran’s *I* index was calculated and local indicators of spatial association (LISA) cluster maps were generated. The global Moran’s *I* index, which ranged from −1 to 1, was calculated firstly to assess the spatial clustering of variables across the study area. Positive spatial autocorrelation was indicated when Moran’s *I* > 0 with a *Z*-score > 1.96, and negative autocorrelation was indicated when Moran’s *I* < 0 with a *Z*-score < −1.96. Subsequently, a LISA analysis was performed, and LISA cluster maps were generated to detect the spatial clustering within specific local areas. The clusters were classified into high–high cluster (HH), high–low cluster (HL), low–low cluster (LL), and low–high cluster (LH). Statistical significance was tested by *Z*-test of LISA metrics, and a *p*-value of <0.05 was considered as a significant local spatial autocorrelation [24].

#### 2.3.2. Generalized Additive Model (GAM)

Given that air pollution data were reported daily while socioeconomic status was reported annually, this study constructed separate models to assess the association between HFMD risk and these two types of variables. To avoid strong correlations and multicollinearity among variables included in the multivariable analysis model, we performed Spearman correlation analysis to assess the correlation between independent variables. When a strong correlation was identified (|r_s_| > 0.80), the variable considered to have greater clinical or epidemiological relevance with incidence based on previous studies was retained. Among the selected variables, variance inflation factor (VIF) was used to evaluate the multicollinearity, and those with VIF > 10 were excluded. In the final GAM, PM_2.5_, O_3_, SO_2_, CO, and NO_2_ were included in the model for air pollution, and GDP per capita, the number of hospital beds per 100,000 population, population density, and proportion of children <15 years were included in the model for socioeconomic status. Multicollinearity testing confirmed that all nine variables could be included in the multivariable analysis (Supplementary Materials Figure S1).

GAM was applied to determine the exposure–response relationships of various and assess cumulative relative risks (RRs) with 95% confidence intervals (CIs). The GAM extends generalized linear models by incorporating additive components where each term represents a nonparametric smooth function describing the relationship between variables and the outcome. This approach shows flexibility in capturing nonlinear associations. To account for potential lagged effects of exposure variables on HFMD incidence, a distributed lag nonlinear model (DLNM) was applied first. Cross-basis functions were constructed for each variable using a fourth-degree polynomial function and a linear function for the exposure–response relationship. Based on the incubation period of HFMD and previous studies, a lag period of 0–7 days was considered for assessing short-term associations between variables and HFMD incidence. The lag structure was stratified by region. These functions were then incorporated as covariates in the GAM, fitted using quasi-Poisson distribution with a log link function. To control for autocorrelation, we included the one-day lagged incidence as a covariate [25]. Given that GAM focused on exposure–response relationships rather than spatial prediction, and that the spatial level of analysis (county) may partially capture regional variation, spatial dependence was not incorporated into the GAM. Model diagnostics and performance evaluation were performed for the model for air pollution using deviance explained, adjusted R-squared, and generalized cross-validation (GCV) score. The mathematical formulation of the GAM is expressed as follows:

$$g(\mu )=s_{0}+s_{1}(X_{1})+s_{2}(X_{2})+\cdots +s_{p}(X_{p})\;\;\;\;\;\;n=s_{0}+\sum_{i=1}^{p}s_{i}(X_{i})$$

g(μ) is the link function; μ = E(Y|X_1_, X_2_, …, X_p_); s_0_ is the intercept; n is the linear predictor; S_i_() is the nonparametric smooth function; and X_i_ is the predictor variable for the smooth function.

All analyses were performed using ArcGIS 10.2 and R software (version 4.4.2).

## 3. Results

### 3.1. Epidemiological Trends of HFMD in Fujian in 2014–2023

**Figure 2:**
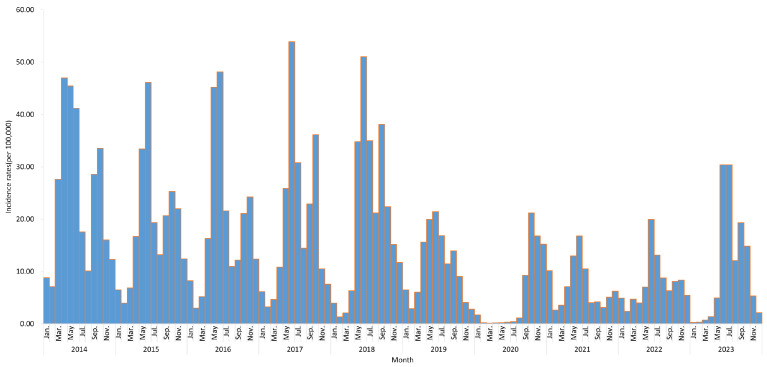
Time series of HFMD incidence rates in Fujian Province from 2014 to 2023.

From 1 January 2014 to 31 December 2023, a total of 672,358 HFMD cases were reported in Fujian Province, including 1092 severe cases and 23 deaths. The average incidence rate was 169.58 per 100,000 and the mortality rate was 0.01 per 100,000. The case fatality rate was 3.45 per 100,000. From 2014 to 2018, the incidence of HFMD remained relatively stable. The incidence began to decline from 2019, with a greater decrease observed between 2020 and 2022. However, in 2023, there was a slight rebound in incidence. Overall, HFMD in Fujian Province showed a distinct seasonality, with two annual peaks. The primary peak occurred between May and June, and the secondary peak appears in September to November (Figure 2).

**Figure 3:**
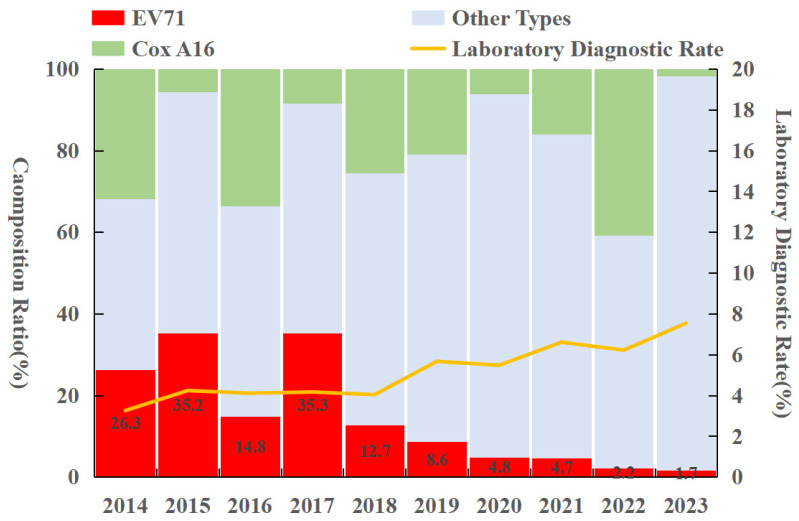
Pathogen profile of HFMD in Fujian Province from 2014 to 2023.

Enterovirus typing was performed using real-time reverse transcription polymerase chain reaction (real-time RT-PCR). A total of 31,342 laboratory-confirmed HFMD cases were reported in Fujian Province from 2014 to 2023, accounting for 4.66% of all reported cases. Among these, EV-A71 was detected in 5097 cases (16.26%), CV-A16 in 5994 cases (19.12%), and other enteroviruses in 20,251 cases (64.62%). Notably, the proportion of EV-A71 cases showed an overall declining trend. CV-A16 and other enteroviruses accounted for higher proportions in 2014, 2016, and 2022, while other enteroviruses predominated in the remaining years (Figure 3).

### 3.2. Spatial Clustering of HFMD

The global spatial autocorrelation analysis showed that Moran’s *I* values were all greater than 0 and statistically significant, indicating a positive spatial autocorrelation of HFMD incidence across Fujian in 2014–2023. These results confirm that the spatial distribution was clustered rather than random. The spatial correlation was weakest in 2014 (Moran’s *I* = 0.16) and strongest in 2020 (Moran’s *I* = 0.37) (Table 1).

**Figure 4:**
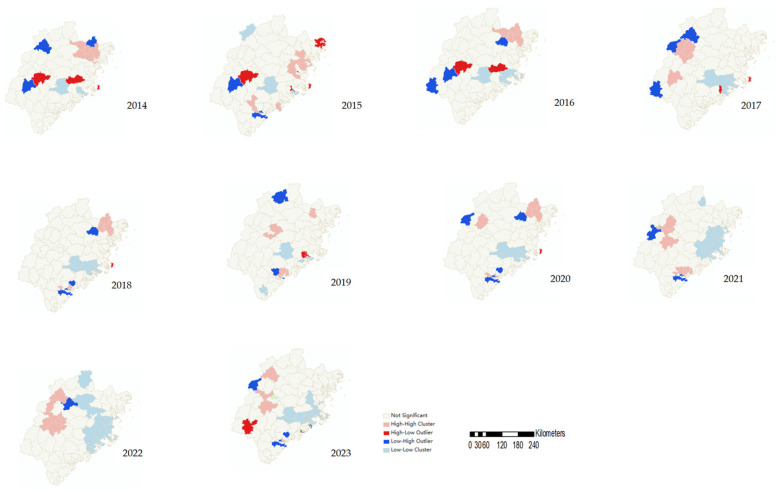
LISA cluster maps of HFMD in Fujian Province from 2014 to 2023.

Local spatial autocorrelation analysis revealed significant high–high clusters (hotspots) and low–low clusters (coldspots) of HFMD incidence in Fujian during 2014–2023. The hotspots were primarily distributed in Ningde, Sanming, and Xiamen, while the coldspots were located in Quanzhou, Fuzhou, and Putian. The spatial clustering patterns varied over time. From 2014 to 2019, hotspots were concentrated in Ningde, and they were predominantly found in Sanming in 2020–2023, with frequent clusters also observed in the Xiang’an and Tong’an districts of Xiamen. Coldspots were relatively stable, consistently located in Yongchun and Dehua counties (Quanzhou), Hanjiang and Chengxiang districts (Putian), and Fuqing county (Fuzhou). Notably, in 2020, the coverage of the coldspot was the largest, encompassing 20 counties across Nanping, Fuzhou, and Quanzhou (Figure 4).

### 3.3. Effects of Environmental and Socioeconomic Determinants on HFMD Risk

**Figure 5:**
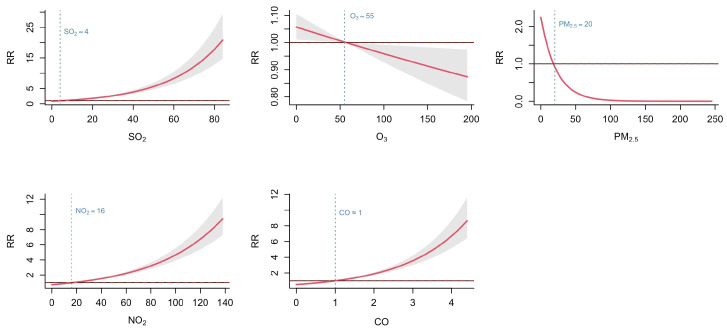
Associations between air pollutants and HFMD in Fujian Province from 2014 to 2023.

The model fitting results showed that the GAM explained 9.35% of the deviance in HFMD incidence, with an adjusted R-squared of 6.39% and a GCV score of 0.415. Although the indexes did not reach ideal levels, it still indicates the independent association between air pollutants and HFMD incidence, given the multifactorial nature of HFMD transmission. Key findings of GAM revealed that the cumulative exposure–response curves for SO_2_, NO_2_, and CO exhibited a monotonically increasing pattern. At concentrations below the median levels (SO_2_ ≈ 4 μg/m^3^, NO_2_ ≈ 16 μg/m^3^, CO ≈ 1 mg/m^3^), the cumulative RR were less than 1, suggesting lower transmission risk at low pollution levels. RR increased progressively with rising pollutant concentrations. The curves for O_3_ and PM_2.5_ showed a decreasing trend. At sub-median concentrations (O_3_ ≈ 55 μg/m^3^, PM_2.5_ ≈ 20 μg/m^3^), the cumulative RRs were greater than 1, implying higher transmission risks in low-to-moderate concentrations. As O_3_ and PM_2.5_ concentrations increased, the RR declined to less than 1 (Figure 5).

**Figure 6:**
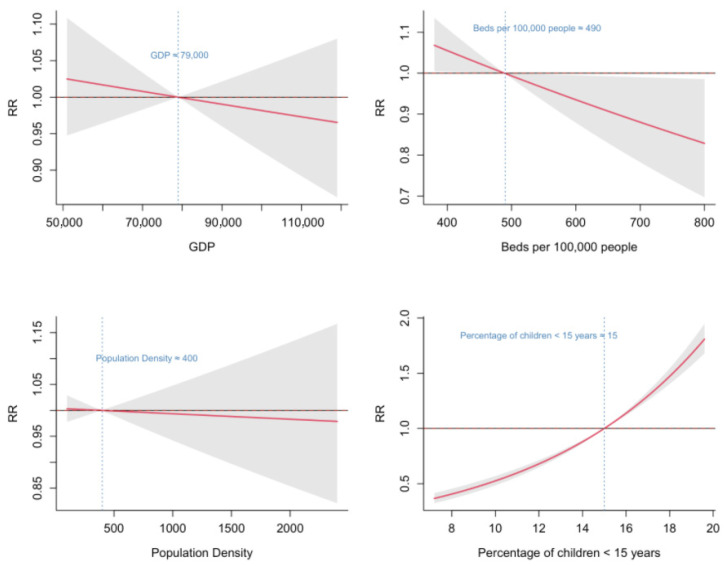
Associations between socioeconomic factors and HFMD in Fujian Province from 2014 to 2023.

Regarding socioeconomic factors, the proportion of children < 15 years and the number of hospital beds per 100,000 population were significantly associated with HFMD incidence. The number of hospital beds per 100,000 population showed a monotonically decreasing relationship with HFMD risk, indicating that the RR of HFMD declined as the number of hospital beds per 100,000 population increased. When the number of beds exceeded 490 per 100,000 population, the RR was less than 1. The proportion of children < 15 years exhibited a monotonically increasing association, with RR exceeding 1 when the proportion surpassed 15%. No significant associations were observed between HFMD incidence and either per capita GDP or population density (Figure 6).

## 4. Discussion

This study identified several key epidemiological characteristics of HFMD in Fujian Province. The disease exhibited distinct seasonal patterns with incidence peaks occurring in late spring and autumn, while the proportion of EV-A71 serotype among causative pathogens showed a significant declining trend. Spatial analysis revealed pronounced geographical clustering of cases, with clearly defined high-risk areas. Environmental risk factor assessment demonstrated that elevated concentrations of SO_2_, NO_2_, and CO were associated with increased HFMD risk, whereas higher levels of O_3_ and PM_2.5_ appeared to have protective effects. Furthermore, we found that regions with higher young populations and regions with lower bed availability face greater transmission risks.

From 2014 to 2023, the reported incidence rate of HFMD in Fujian Province was 168.09 per 100,000 population, which was higher than the national average level (147.82 per 100,000 from 2011 to 2018) [26]. Since 2019, the incidence of HFMD has showed a significant downward trend. This decline may be partly attributed to improvements in socioeconomic conditions, living environments, sanitation, and public health interventions [2]. Additionally, it may also be related to the implementation of control measures and heightened public awareness of infectious disease prevention during the COVID-19 pandemic. The disease exhibited distinct seasonal patterns in Fujian, with a primary peak in May–June and a secondary peak in September–November, consistent with southern China’s epidemiological profile [27]. The peak incidence periods occur during the transition between spring and summer, and between autumn and winter in these regions. This pattern may be associated with greater diurnal temperature variations in seasonal changes, which can weaken the immune system, or with increased indoor activities that promote gathering and facilitate transmission. Notably, Fujian’s low severe-case and mortality rates may reflect the declining prevalence of EV-A71, the primary causative agent of severe HFMD [4]. However, the proportion of other enteroviruses has risen significantly in recent years, warranting heightened vigilance. These alternative strains nonetheless pose serious risks, including severe complications and fatal outcomes [28,29]. Notably, EV-D68 caused outbreaks in North America and Europe, resulting in at least 14 documented pediatric deaths [30,31,32]. This underscores the urgent need for expanded surveillance and research on emerging enterovirus variants associated with HFMD. This epidemiological shift necessitates enhanced surveillance and differentiation of emerging enterovirus serotypes. Public health authorities must maintain vigilance against potential outbreaks caused by these alternative strains and implement effective prevention and control measures to mitigate viral transmission.

Wang et al. [33] employed spatiotemporal scanning methods to analyze HFMD case clustering at the county level across China, revealing significant aggregation in eastern and southern regions—including Fujian Province, located in the country’s southeastern area. Our study similarly identified spatial clustering of HFMD cases in Fujian, with high–high clusters primarily concentrated in Ningde, Sanming, and Xiamen. Notably, these high-incidence areas exhibit substantial disparities in environmental conditions and socioeconomic development. As a common infectious disease, HFMD transmission is influenced by multifaceted factors, including geographical [34], environmental [11], and socioeconomic determinants [18]. Therefore, it is essential to incorporate spatial clustering with geographic environmental factors and socioeconomic determinants to comprehensively characterize the disease transmission patterns.

Previous studies have explored the relationship between air pollution and HFMD incidence, though findings remain inconsistent. Lin et al. proposed that elevated particulate matter levels may facilitate viral transmission by enhancing viral attachment to airborne particles [35]. Conversely, Bo et al. suggested that air pollutants could compromise host immunity, thereby increasing susceptibility to infection [36]. These contrasting mechanisms highlight the complex interplay between environmental factors and enterovirus epidemiology. Our study revealed distinct dose–response relationships between specific air pollutants and HFMD incidence in Fujian. Increasing concentrations of SO_2_, NO_2_, and CO were associated with a progressively elevated disease risk, aligning with their known pro-inflammatory effects. However, the risk of HFMD incidence gradually decreased with rising levels of O_3_ and PM_2.5_, which differs from the findings of some previous studies [12,13,14,36]. One possible explanation is the spatial heterogeneity in the relationship between air pollutants and HFMD risk. The same pollutant may present different effects in different regions. These associations may also be modified by factors such as climatic conditions or other environmental determinants. Another explanation is that O_3_ and PM_2.5_ are positively correlated in southern China [37], with high levels of PM_2.5_ often accompanied by elevated concentrations of O_3_. People may engage in risk-reducing behaviors during periods of poor air quality, such as limiting children’s outdoor activities. Additionally, the strong oxidative properties of O_3_ could contribute to viral inactivation in the environment.

The study found that the proportion of younger age groups had a significant effect on HFMD incidence, aligning with the well-established epidemiological pattern that HFMD primarily affects children under five years of age [2]. This highlights the importance of prioritizing children under 15 years old in HFMD prevention and control strategies. Also, we observed that HFMD risk declined as the number of hospital beds per 100,000 population increased, consistent with the findings of Jiang et al. [18]. The availability of hospital beds reflects the development and capacity of healthcare system. Regions with greater bed density are likely to provide better diagnosis, treatment, and prevention services for HFMD, potentially reducing the transmission risk of HFMD. However, in contrast to the studies by Li et al. [20] and Gou et al. [21], we found no significant associations between HFMD incidence and either per capita GDP or population density. This discrepancy may be explained by two reasons. First, the socioeconomic data were available only at an annual scale, and the coarse temporal resolution may obscure relationships with HFMD incidence. Second, the effects of socioeconomic variables might be confounded or offset by other environmental or behavioral factors. Further research with monthly socioeconomic data or applying calibration methods is needed to clarify the effects.

### Limitations

This study has several limitations. First, the incidence data were derived from official surveillance records, which may underestimate the true burden of HFMD due to potential underreporting and inadequate healthcare-seeking behavior among patients [38]. Second, socioeconomic variables were only available at an annual scale instead of monthly or daily data, which has limited the capacity to capture finer-scale associations between these factors and disease transmission. Third, although this study analyzed the spatial patterns of HFMD distribution, spatial factors were not explicitly incorporated into the analytical model of influencing factors. Future studies should consider incorporating additional variables, such as meteorological conditions, population mobility, and spatiotemporal factors, to gain a more comprehensive understanding of HFMD transmission dynamics.

## 5. Conclusions

The incidence of HFMD in Fujian Province is significantly higher than the national average, exhibiting distinct seasonal patterns and significant spatiotemporal clustering. Increased concentrations of SO_2_, NO_2_, and CO were associated with elevated HFMD risk raised, while higher levels of O_3_ and PM_2.5_ showed protective effects. Among socioeconomic factors, the HFMD risk increased with a higher proportion of children under 15 years and with a smaller number of hospital beds per 100,000 population. We recommend establishing a comprehensive multipollutant monitoring and early warning system in high-risk areas to facilitate the early detection of potential environmental health threats. It should integrate multisource data and intelligent analysis to enable the coordinated surveillance of various pollutants in air, water, and other environmental media. Furthermore, differentiated public health response strategies should be tailored to the specific characteristics, sources, and health impacts of air pollutants to reduce population exposure risks. Additionally, infectious disease prevention and control measures should be strengthened in settings with high concentrations of children, including routine morning health screenings in schools and improved ventilation and disinfection in children’s recreational facilities. This multifaceted intervention approach may substantially enhance the effectiveness of HFMD prevention and control efforts.

## Figures and Tables

**Table 1:** Global Moran’s *I* values of HFMD in Fujian Province from 2014 to 2023.

Year	Moran’s *I*	*Z*-Score	*p*-Value
2014	0.16	2.34	0.02
2015	0.21	3.10	0.00 *
2016	0.17	2.51	0.01
2017	0.17	2.57	0.01
2018	0.20	2.92	0.00 *
2019	0.36	5.24	0.00 *
2020	0.37	5.36	0.00 *
2021	0.21	3.09	0.00 *
2022	0.34	4.95	0.00 *
2023	0.25	3.41	0.00 *

*: *p* < 0.001.

## Data Availability

The original contributions presented in this study are included in the article/Supplementary Materials. Further inquiries can be directed to the corresponding author.

## Supplementary Materials

The following supporting information can be downloaded at: https://www.mdpi.com/article/10.3390/tropicalmed10070188/s1, Figure S1. Correlation Between Air Pollutants and Socioeconomic Factors in Fujian Province, China (2014–2023).
