# Supplementary material for: Spatiotemporal Distribution of Hand, Foot, and Mouth Disease and the Influence of Air Pollutants and Socioeconomic Factors on Incidence in Fujian, China
Source: Trop Med Infect Dis. 2025 Jul 3;10(7):188. doi: 10.3390/tropicalmed10070188 (PMC12298525; doi:10.3390/tropicalmed10070188)
Supplement: Supplementary file 1 [file tropicalmed-10-00188-s001.zip › tropicalmed-3616724-supplementary.pdf]

Supplementary Materials

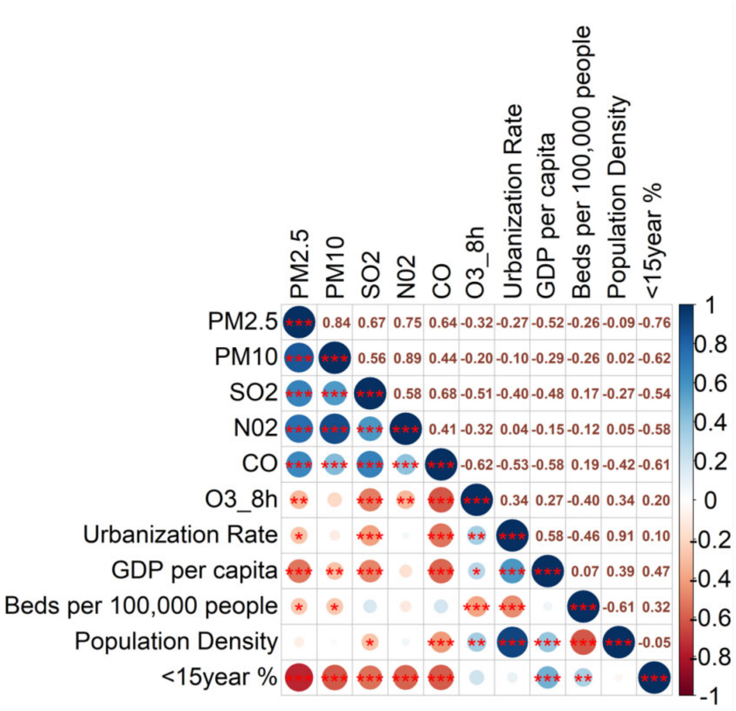

Figure S1. Correlation Between Air Pollutants and Socioeconomic Factors in Fujian Province, China (2014-2023)
